# Performance of Lactate and CO_2_-Derived Parameters in Predicting Major Postoperative Complications After Cardiac Surgery With Cardiopulmonary Bypass: Protocol of a Diagnostic Accuracy Study

**DOI:** 10.3389/fcvm.2021.724713

**Published:** 2021-10-01

**Authors:** Xiao-Fen Zhou, Rong-Guo Yu, Qian Chen, Yi-Min Xue, Han Chen

**Affiliations:** Department of Critical Care Medicine, Shengli Clinical Medical College of Fujian Medical University, Fujian Provincial Hospital, Fuzhou, China

**Keywords:** lactate, Pv-aCO_2_/Ca-vO_2_, postoperative complications, cardiopulmonary bypass, diagnostic accuracy study

## Abstract

**Background:** CO_2_-derived parameters are increasingly used to identify either low-flow status or anaerobic metabolism in shock resuscitation. However, the performance of CO_2_-derived parameters in cardiac surgical patients is poorly understood. This study aims to compare the performance of lactate and CO_2_-derived parameters in predicting major postoperative complications after cardiac surgery with cardiopulmonary bypass.

**Methods:** This is a prospective, single-center, diagnostic accuracy study. All patients who receive elective cardiac surgery involving cardiopulmonary bypass will be screened for study eligibility. Blood samples will be taken for the calculation of CO_2_-derived parameters, including the venous-arterial difference in CO_2_ partial pressure (PCO_2_ gap), venous-arterial difference in CO_2_ content to arterial-venous O_2_ content ratio (Cv-aCO_2_/Ca-vO_2_), and venous-arterial difference in CO_2_ partial pressure to arterial-venous O_2_ content ratio (Pv-aCO_2_/Ca-vO_2_) at ICU admission, and 3, 6, and 12 h later. Baseline, perioperative data will be collected daily for 7 days; patients will be followed up for 28 days to collect outcome data. The primary endpoint is the occurrence of major postoperative complications. Receiver-operating characteristics (ROC) curve analysis will be carried out to assess the predictive performance of lactate and CO_2_-derived parameters. The performance of the ROC curves will be compared.

**Discussion:** The performance of lactate and CO_2_-derived parameters in predicting major postoperative complications will be investigated in the non-sepsis population, which has not been extensively investigated. Our study will compare the two surrogates of respiratory quotient directly, which is an important strength.

**Trial Registration:** ChiCTR, ChiCTR2000029365. Registered January 26th, 2020, http://www.chictr.org.cn/showproj.aspx?proj=48744.

## Background

Complications after cardiac surgery (e.g., acute respiratory failure, circulatory failure, acute kidney injury, neurological failure, etc.) are associated with high morbidity and mortality (1–3). One crucial cause is the mismatch of oxygen delivery and consumption after the surgery, which is often the result of a decrease in cardiac output and/or an increase in oxygen demand due to stress (4, 5). The imbalance of oxygen delivery and consumption leads to tissue hypoxia. Organ injury and dysfunction can occur if tissue hypoxia is not corrected. Timely identification and management of tissue hypoxia are essential to prevent the development of organ dysfunction and postoperative complications. Several biomarkers of hypoxia have been proposed to identify tissue hypoxia (6–8).

Lactate is the most widely adopted biomarker of tissue hypoxia. The occurrence rate of hyperlactatemia is ~10–20% in patients receiving cardiac surgery (9). It has long been known that decreasing oxygen delivery to the tissues increases serum lactate levels. However, as lactate is a normal end product of glucose metabolism, it may increase due to many other causes rather than hypoxia. Besides, it has been suggested that hyperlactatemia has a bimodal distribution in the perioperative period: an early-onset hyperlactatemia strongly suggests tissue ischemia and is associated with a poor outcome; in contrast, a late-onset hyperlactatemia is not caused by an impairment of tissue perfusion and has a more benign course (10). Therefore, the efficiency of this marker is still questioned, although the association between an elevated lactate level and adverse outcomes after cardiac surgery has been confirmed in several studies (9, 11).

Recently, a series of CO_2_-derived parameters have attracted both clinicians' and researchers' attention (12–15). Current examinations are not satisfactory enough to answer the question of adequacy between oxygen supply and demand. Lactate, as previously discussed, is a sensitive marker of anaerobic metabolism, but with many false positives. Urine output, at its best, reflects the function of one organ only. The mixed venous oxygen saturation (SvO_2_) and the central venous oxygen saturation (ScvO_2_) reflect the oxygen exertion rate. However, they are often in a normal range or even in a supra-normal level in septic shock despite the presence of tissue anaerobic metabolism, partly due to microcirculatory shunting or the inability of the tissues to use oxygen (i.e., cytopathic hypoxia), or both (16, 17). CO_2_-derived parameters overcome many of the limitations of the previous indices in indicating anaerobic tissue metabolism. The underlying physiology rationales are discussed in detail elsewhere (18); one can briefly interpret the concept as follows: the venous-arterial difference in CO_2_ partial pressure (Pv-aCO_2_ or PCO_2_ gap) is a useful index of the adequacy of cardiac output (CO) for the global metabolic conditions. In other words, a decrease in CO results in an increased PCO_2_ gap and *vice versa*. However, it fails when it serves as a marker of tissue hypoxia and anaerobic metabolism (19). In contrast, the venous-arterial difference in CO_2_ content to arterial-venous O_2_ content ratio (Cv-aCO_2_/Ca-vO_2_, which is a surrogate of the respiratory quotient) could serve as a marker of global anaerobic metabolism (20). Due to the complexity of the calculation of Cv-aCO_2_, the formula is “simplified” by replacing Cv-aCO_2_ with PCO_2_ gap, since the relationship between Cv-aCO_2_ and PCO_2_ gap is almost linear over the physiological range (Pv-aCO_2_ = κ × Cv-aCO_2_). Thus, Cv-aCO_2_ can be estimated at the bedside by PCO_2_ gap, where the κ is the factor defining the relation between CO_2_ partial pressure and CO_2_ content.

The above-mentioned CO_2_-derived parameters are increasingly used to identify either low-flow status (PCO_2_ gap) or anaerobic metabolism (Cv-aCO_2_/Ca-vO_2_ and Pv-aCO_2_/Ca-vO_2_) in shock resuscitation, particularly in septic shock (21, 22). However, they are not extensively investigated in cardiac surgery patients. Most studies focused on the predictive value of the PCO_2_ gap (23–26). Some other studies took the Pv-aCO_2_/Ca-vO_2_ into account as well (12, 27, 28). However, Cv-aCO_2_/Ca-vO_2_ was not measured in those studies. This study aims to compare the performance of lactate and CO_2_-derived parameters (i.e., PCO_2_ gap, Pv-aCO_2_/Ca-vO_2_, and Cv-aCO_2_/Ca-vO_2_) in predicting major postoperative complications after cardiac surgery with cardiopulmonary bypass.

## Methods

### Study Design Overview

The present study is a prospective, single-center, diagnostic accuracy study involving patients undergoing elective cardiac surgery. The study will be conducted in Fujian Provincial Hospital, a tertiary hospital and a teaching hospital of Fujian Medical University.

### Ethical Approval and Informed Consent

The study protocol and consent forms were approved on January 21st, 2020, by the Ethics Committee of Fujian Provincial Hospital (Approval #K2020-01-022). The study was registered on January 26th, 2020, at the Chinese Clinical Trial Registry (ChiCTR2000029365). Details including the items from the World Health Organization Trial Registration Data Set can be found on http://www.chictr.org.cn/showproj.aspx?proj=48744. Protocol amendments (if any) will be approved by the Ethics Committee.

A study coordinator will be introduced to the family after the eligibility screen. The ICU physician will ensure that the family is aware of the study coordinator's credentials. The study coordinator will describe every relevant aspect of the project. Meanwhile, the study coordinator will frequently pause to ask if there is any question or request and ask the family to repeat the topic being discussed in their own words to ensure that they understand. It will be emphasized that the participants are free to decline consent without consequences and that they can withdraw consent at any time. Written consent will be obtained in the presence of a witness.

### Formulas and Calculation

According to Fick's equation, we have:

(1) $$\begin{array}{r} \text{V}{\text{O}}_{2}=\text{CO}\times \left(\text{Ca}{\text{O}}_{2}-\text{Cv}{\text{O}}_{2}\right) \end{array}$$

(2) $$\begin{array}{r} \text{VC}{\text{O}}_{2}=\text{CO}\times \left(\text{CvC}{\text{O}}_{2}-\text{CaC}{\text{O}}_{2}\right) \end{array}$$

where VO_2_ is O_2_ consumption, CO is cardiac output, CaO_2_ is arterial O_2_ content, CvO_2_ is venous O_2_ content, VCO_2_ is CO_2_ production, CaCO_2_ is arterial CO_2_ content, CvCO_2_ is venous CO_2_ content.

From Equations (1) and (2), we have:

$$\begin{array}{l} \frac{{\text{VCO}}_{2}}{{\text{VO}}_{2}}=\frac{\left({\text{CvCO}}_{2}-{\text{CaCO}}_{2}\right)}{\left({\text{CaO}}_{2}-{\text{CvO}}_{2}\right)} \end{array}$$

VCO_2_/VO_2_ represents the respiratory quotient, and therefore, the right part of the equation (Cv-aCO_2_/Ca-vO_2_) serves as a surrogate of the respiratory quotient.

The following formula can describe the relationship between CO_2_ partial pressure and CO_2_ content:

(3) $$\begin{array}{r} \text{Pv-aC}{\text{O}}_{2}=k\times \left(\text{CvC}{\text{O}}_{2}-\text{CaC}{\text{O}}_{2}\right) \end{array}$$

Combined with Equation (2), we have:

$$\begin{array}{l} \text{VC}{\text{O}}_{2}=\text{CO}\times \left(\text{PvC}{\text{O}}_{2}-\text{PaC}{\text{O}}_{2}\right)/k \end{array}$$

Rearrange:

$$\begin{array}{l} \left(\text{PvC}{\text{O}}_{2}-\text{PaC}{\text{O}}_{2}\right)=\left(\text{VC}{\text{O}}_{2}\times k\right)/\text{CO} \end{array}$$

This relationship between CO and PCO_2_ gap expresses that, to a given VCO_2_ and κ (which will not dramatically change in a short period), the PCO_2_ gap inversely correlates with CO.

The O_2_ related variables are calculated as follows:

- CaO_2_ = (Hb × SaO_2_ × 1.34) + (PaO_2_ × 0.0031)
- CvO_2_ = (Hb × SvO_2_ × 1.34) + (PvO_2_ × 0.0031)

CO_2_ contents is calculated according to the Douglas formula (29):

- Blood CO_2_ content = plasma CO_2_ content × (1 – 0.0289 × Hb) ÷ (3.352 – 0.456 × SO_2_) × (8.142 – pH)
- Plasma CO_2_ content = 2.226 × plasma CO_2_ solubility × plasma PCO_2_ × (1 + 10^pH−pK′^)
- Plasma CO_2_ solubility = 0.0307 + 0.00057 × (37 – T) + 0.00002 × (37 – T)^2^
- pK′ = 6.086 + 0.042 × (7.4 – pH) + (38 – T) × [0.00472 + 0.00139 × (7.4 – pH)]

### Study Setting and Population

The study setting is the cardiac intensive care unit (ICU) (20 beds) and the department of cardiac surgery (118 beds) at a tertiary teaching hospital.

All patients who receive elective cardiac surgery involving cardiopulmonary bypass (CPB) will be screened for study eligibility.

The inclusion criteria are (1) Age ≥ 18; (2) Receive elective cardiac surgery involving CPB; (3) Admitted into ICU after the surgery; (4) With an arterial line and a central venous catheter in place. Exclusion criteria are: (1) Acute or chronic kidney disease prior to surgery (2) Acute or chronic hepatic insufficiency prior to surgery; (3) Malpositioning of the central venous catheter (4) History of alcohol abuse; (5) Pregnant; (6) Unwilling to provide consent.

### CPB and Perioperative Management

The induction will consist of propofol, etomidate, or ketamine as appropriate. Patients will be intubated, and a central venous catheter will be placed *via* the internal jugular vein or subclavian vein. The initial tidal volume will be ~8 mL/kg, and the respiratory rate will be adjusted to maintain an arterial CO_2_ pressure (PaCO_2_) of 35–40 mmHg during the surgery. Sufentanil or remifentanil will be used for analgesia. The neuromuscular blockade will be induced by using rocuronium, cisatracurium or atracurium. Continuous administration of propofol or sevoflurane will be used for anesthesia maintenance. After attaining an activating clotting time (ACT) > 480 s *via* heparin administration, CPB will be initiated by using an occlusive roller pump (Jostra, Germany) and a membrane oxygenator (Affinity 7,000, USA). The pump flow will be around 2.0–2.6 L/min/m^2^ during CPB. The mean arterial pressure (MAP) will be maintained at 60–80 mmHg. Cephazolin will be administered as perioperative antibiotic prophylaxis. Patients will be transmitted to and monitored in the ICU after the surgery.

The postoperative ICU management will be as follows: fluid infusion (normal saline, Ringer's solution, or gelatin); red blood cells will be transfused to maintain hemoglobin concentration; fresh frozen plasma and platelets will be given when bleeding was suspected; vasopressors and inotropes will be continuously infused to achieve the hemodynamic targets: MAP ≥65 mmHg, urine output ≥0.5 mL/kg, cardiac index ≥2.5 L/min/m^2^ or a mixed venous saturation of >60%; analgesics will include sufentanil or remifentanil; sedation will consist of propofol or midazolam; ventilation will be continued until patients are awake, hemodynamic stable and with no bleeding, after that patients will be wean from the ventilator and extubated.

### Data Collection and Follow-Up

Once admitted into ICU, demographic data, history of past illness, diagnosis, preoperative ejection fraction, and the New York Heart Association (NYHA) functional classification will be collected. The type of surgery, duration of operation, CPB and aortic clamping, intra-operative transfusion, net fluid balance, and the doses of vasopressors and inotropic agents during the operation will also be recorded. The sequential organ failure assessment score (SOFA) and Acute Physiology and Chronic Health Evaluation (APACHE) II score at enrollment and the daily highest score during the follow-up period will be recorded. Blood samples will be drawn *via* both arterial line and central venous catheter for blood gas analysis (GEM 3500, Instrumentation Laboratory Co., MA, USA) at ICU admission (T_0_), and 3 (T_3_), 6 (T_6_), and 12 h (T_12_) later. No personal information will be collected.

Patients will be followed up for 28 days. The occurrence of major postoperative complications (see below) will be recorded. The duration of mechanical ventilation and length of ICU stay will also be recorded. Daily fluid balance and the highest vasoactive-inotropic score (VIS) will be calculated. VIS will be calculated as dopamine dose (μg/kg/min) + dobutamine dose (μg/kg/min) + 100 × epinephrine dose (μg/kg/min) + 100 × norepinephrine dose (μg/kg/min) + 15 × milrinone dose (μg/kg/min) + 10,000 × vasopressin dose (U/kg/min) (30).

### Study Endpoints

The primary endpoint is the occurrence of major postoperative complications, defined according to The European Society of Anesthesiology (ESA) and the European Society of Intensive Care Medicine (ESICM) guideline (31) and Society of Thoracic Surgeons (STS) guidelines (32–34) on perioperative outcome measures. Thus we define major postoperative complications as the occurrence of any of the following: (1) acute kidney injury (AKI) stage 2 or more according to the KDIGO definition (35); (2) acute respiratory distress syndrome (ARDS) according to the Berlin definition (36) or respiratory failure (PaO_2_/FiO_2_ <300 mm Hg); (3) stroke, defined as an embolic, thrombotic or hemorrhagic cerebral event with persistent residual motor, sensory or cognitive dysfunction; (4) delirium identified using the Intensive Care Delirium Screening Checklist (37); (5) myocardial infarction: post-surgical myocardial infarction will be screened if serum cardiac biomarker (troponin I) continues to increase after surgery, or decreases and then increases again; and diagnosis will be confirmed if (i) new pathological Q waves appear in different territories than those identified before surgery, or (ii) angiographically documented new native coronary artery occlusion, or (iii) imaging evidence of new loss of viable myocardium or new regional wall motion abnormality (38); (6) circulatory shock for any cause, defined as follows: (i) systolic arterial pressure <90 mm Hg or the mean arterial pressure <70 mm Hg, or blood pressure decreases by >40 mm Hg from baseline, and (ii) clinical signs of tissue hypoperfusion, and iii) serum lactate >2 mmol/L (39); (7) severe sepsis or septic shock according to the Surviving Sepsis Guidelines definition (40); (8) abdominal compartment syndrome: intra-abdominal pressure >20 mm Hg; (9) reoperation; (10) cardiac arrest; (11) death from any cause.

The secondary endpoints include the duration of mechanical ventilation, length of ICU stay, ICU mortality, and hospital mortality. Data will be collected and analyzed from all patients, even if they cannot finish the full-term follow-up (e.g., patients die or withdraw from medical care).

### Sample Size

The incidence of major postoperative complications in a recent study with a similar definition was 56.5% (12). Considering this incidence rate, the variables used in sample size calculation are a ratio of sample sizes in negative/positive groups of 1, an area under the receiver-operating characteristics (ROC) curve of 0.7, a type I error probability (α) of 0.05, a type II error probability (β) of 0.1, and a null hypothesis value of 0.5. By using the MedCalc Statistical Software (Ver. 15.8, MedCalc Software Ltd., Ostend, Belgium), we calculated that 41 patients must be enrolled in each group (i.e., with or without major postoperative complications). The sample size extends to 102 patients to anticipate a drop-out rate of 20% due to missing values.

### Statistical Analysis

EpiData (ver. 3.1, Denmark) will be used for data entry and data quality control. Multiple imputations will be used to handle missing data. Perioperative characteristics will be compared according to the occurrence or not of major postoperative complications. Continuous variables will be assessed for normal distribution and presented as means and standard deviations or medians and inter-quartile ranges as appropriate; the inter-group comparison will be performed by using Student's *t*-test for normally distributed variables or Mann-Whitney *U*-test for non-normally distributed variables. Categorical variables will be presented as numbers and percentages and analyzed by using χ^2^-test or Fisher's exact tests as appropriate. Ordinal variables will be analyzed by using the rank-sum test.

ROC curve analysis will be carried out to assess the predictive performance of lactate and CO_2_-derived parameters. The optimal thresholds for predicting major postoperative complications will be determined by maximizing the Youden index. Sensitivity, specificity, predictive values, and likelihood ratios will be calculated. The performance of the ROC curves will be compared by using DeLong's method (41). A *p* < 0.05 (two-tailed) will be considered significant. Analyses will be performed by using SPSS Statistical Software (Ver. 23.0, IBM Co., NY, USA) and MedCalc Statistical Software (for the comparison of ROC curves).

### Data Management and Dissemination of Results

Only members of the research team will have access to the research data. All data collected will be kept in a patient file and stored in a locker. At the completion of the study, all case report forms will be collated and archived. All electronic files will be password protected. All patient files (hard copy and electronic) will be handled consistent with the regulations of the hospital regarding the retention and disposal of patient records. No interim analyze is planned. The results of this study will be submitted to an international peer-reviewed journal. The results will also be disseminated via national and international scientific meetings. The datasets obtained from the present study will be available from the corresponding author on reasonable request.

## Discussion

Although CO_2_-derived parameters are increasingly used to identify either low-flow status and/or anaerobic metabolism in shock resuscitation, they are not extensively investigated in cardiac surgery patients. Most studies focused on the predictive value of the PCO_2_ gap (23–26). Some other studies took the Pv-aCO_2_/Ca-vO_2_ into account as well (12, 27, 28). However, Cv-aCO_2_/Ca-vO_2_ was not measured in those studies. Although both considered as a surrogate of the respiratory quotient, it is unclear whether Pv-aCO_2_/Ca-vO_2_ is interchangeable with Cv-aCO_2_/Ca-vO_2_ because the relationship between CO_2_ partial pressure and CO_2_ content is not perfectly linear and is influenced by the degree of metabolic acidosis (42, 43), which is a commonly seen situation after cardiac surgery. Our study will compare these two respiratory quotient surrogates directly, which is an important strength.

There are also limitations of the study. First, this study uses a single-center study design, which limits the external validity of the results. Second, central venous rather than mixed venous blood samples will be taken for the calculation of all the CO_2_-derived parameters. Third, although one important purpose of the study is to compare the performance of the surrogates of the respiratory quotient, no “gold-standard” method will be used for the measurements of the respiratory quotient.

## Trial Status

Protocol version number and date: version 1.0, January 21st, 2020. Recruitment is not started yet, and it is expected to continue until June 2023 to complete patient recruitment.

## Ethics Statement

The studies involving human participants were reviewed and approved by the Ethics Committee of Fujian Provincial Hospital (Approval # K2020-01-022). The patients/participants provided their written informed consent to participate in this study.

## Author Contributions

All authors edited the manuscript and approved the final manuscript.

## Funding

This study received funding from Fujian Puruikang Medical Equipment Co., Ltd. HC was supported by the High-level Hospital Foster Grants from Fujian Provincial Hospital (Grant Number: 2020HSJJ02). The funders were not involved in the study design, collection, analysis, interpretation of data, the writing of this article, or the decision to submit it for publication.

## Conflict of Interest

The authors declare that the research was conducted in the absence of any commercial or financial relationships that could be construed as a potential conflict of interest.

## Publisher's Note

All claims expressed in this article are solely those of the authors and do not necessarily represent those of their affiliated organizations, or those of the publisher, the editors and the reviewers. Any product that may be evaluated in this article, or claim that may be made by its manufacturer, is not guaranteed or endorsed by the publisher.

## Abbreviations

AKI: acute kidney injury
ARDS: acute respiratory distress syndrome
APACHE II: Acute Physiology and Chronic Health Evaluation II
CO: cardiac output
CPB: cardiopulmonary bypass
CaO_2_: arterial O_2_ content
CaCO_2_: arterial CO_2_ content
CvO_2_: venous O_2_ content
CvCO_2_: venous CO_2_ content
Cv-aCO_2_/Ca-vO_2_: venous-arterial difference in CO_2_ content to arterial-venous O_2_ content ratio
ESA: European Society of Anesthesiology
ESICM: European Society of Intensive Care Medicine
ICU: intensive care unit
MAP: mean arterial pressure
NYHA: New York Heart Association
PCO_2_ gap: venous-arterial difference in CO_2_ partial pressure
Pv-aCO_2_/Ca-vO_2_: venous-arterial difference in CO_2_ partial pressure to arterial-venous O_2_ content ratio
SOFA: sequential organ failure assessment score
STS: Society of Thoracic Surgeons
SvO_2_: mixed venous oxygen saturation
ScvO_2_: central venous oxygen saturation
VCO_2_: CO_2_ production
VIS: vasoactive-inotropic score.
